# Fatal Mistakes

**Published:** 1850-10

**Authors:** 


*Fatal Mistakes.*—A number of instances have recently occurred in which, by mistakes of apothecaries in putting up wrong medicines, life has been sacrificed. In a recent instance at Boston, Corrosive Sublimate was sent instead of Calomel, as prescribed, and the dose of ten grains was taken by the patient, with a fatal result, as is supposed, and the apothecary has been arrested for manslaughter. His conviction and punishment, however, will not restore the victim to life; but it will not be in vain, if it shall lead to measures which may prevent the repetition of similar mistakes.

Upon this and the like cases, we remark that there are in all our cities numerous drug stores kept by parties who are wholly ignorant of the business, and dependent on drug clerks whom they employ, and who often are mere boys, knowing little more than their employers. These stores are ever changing hands, and the new proprietors go into them to learn the business of preparing and selling medicines, having often had no previous knowledge of drugs. That blunders, which may or may not be fatal, must occur under such circumstances, must be obvious.

Again: In many drug stores the business of putting up prescriptions is very carelessly done—the most inexperienced clerks being allowed to attend to it; which is another source of mistakes.

The remedy for these sources of the mischief lies first with the civil authorities, who ought to protect the health and lives of the community, by prohibiting ignorant and unqualified persons from selling drugs, or doing business as apothecaries, and this under heavy penalties. The popular outcry against monopolies ought not to prevent legislation on so important a subject as the dispensation of drugs; for there can be no doubt that there are but few detections of the fatal mistakes made by such persons as those here alluded to. And yet all these might be prevented by empowering the College of Pharmacy, or some other competent authority, to examine and license all persons claiming to keep apothecary shops, or to dispense medicines of any kind.

But a second remedy lies with the physicians, who should direct all their prescriptions to be procured of some competent apothecary, known to be such. While a third remedy is in the hands of the people themselves, who should in no case purchase medicines except at some reputable store, the proprietor of which is known to have been educated to the business, and reliable from his character and habits.

But there is another aspect of this subject which demands grave consideration by our profession. It is said that mistakes are often made by reason of the loose and careless manner in which prescriptions are written, often scarcely legible, and so abbreviated as to be read with difficulty even by well instructed apothecaries. This matter should be reformed altogether, and will in part remedy the evil.

But still another view of the subject has been taken by the public press, which is worthy of consideration. From time immemorial our profession have written their prescriptions in *Latin*, and employed technicalities in this language in writing their prescriptions. In former days, when a knowledge of the Latin tongue was universally possessed by all physicians and apothecaries, the safety of this course was unquestionable. *Sed tempora mutantur, et nos mutamur cum illis.* In these days of *progress*, when the discovery has been made by "Young Physic" that doctors and apothecaries have no need of Latin, or any other dead language; and that men can do very well in either capacity without a knowledge of even their mother tongue; and when even bold ignorance is often the passport to popularity in the art of healing, a change would seem to be demanded by "the spirit of the age."

It is now urged so clamorously, that even medical authority has been invoked, and has responded affirmatively, that all our prescriptions should be written in the English language, and so plainly as to preclude all possibility of mistake in the article or quantity ordered. And it may have become necessary thus to conform to the degenerate days which have overtaken us, when scholarship is no longer to characterize the profession. Our old physicians will find it awkward and unseemly for a time; since they have been trained in a different school, and at a time when no prescription was ever written in English by any regularly bred physician—for all such were required to write in good medical Latin; and none but those who had entered the profession by climbing over the wall, or in some other clandestine way, were ignorant of this language. And the same might then be said of all reputable apothecaries; for in those days, a knowledge of Latin and Greek was deemed an essential pre-requisite to reputable standing in either department.

But it is not so now; " 'tis true a pity, and pity 'tis, 'tis true." Hence, a necessity would seem to be laid upon us all, to write all our prescriptions so that any ignorant apothecary may read and prepare them. This has been deemed, heretofore, the only protection we had against falling into the hands of ignoramusses in the business; for by the use of Latin technicals we have sought to secure the attention of qualified persons to put up our prescriptions: but this must be relinquished, it seems, and in obedience to "manifest destiny."

Be it so: but will this prevent fatal mistakes, such as those which have recently occurred? Suppose in the case at Boston, "*Calomel* 10 *grains*," had been written, instead of "*Sub Mur. Hydrarg. grs. X.*" Does anybody believe that the mistake of sending half a scruple of *Corrosive Sublimate* would not have occurred precisely as it did? The apothecary either knew better, or he did not; for no intentional wrong is alledged. If he did not know what the receipt called for, how came he to be in an apothecary shop in any capacity? If he did know any better, then he is inexcusable, for the prescription in this case was correctly written. By the *old* nomenclature, calomel was called a "Sub-Muriate of Mercury;" and corrosive sublimate, either a "Muriate," or an "Oxy-Muriate." By the *new* nomenclature, the former is written "Proto-Chloride," and the latter "Per-Chloride;" and these terms ought to be familiar as household words to any tyro in a druggist's store. That they are not so is a shame and disgrace, though, as in this case, so in others, the mistakes are not always to be ascribed to ignorance, but to haste, sheer carelessness, or a recklessness of human life. To write prescriptions in English will not remedy the mischief, though it will remove all pretext for blaming the profession.—*N. Y. Med. Gazette.*

